# Magnesium impairs *Candida albicans* immune evasion by reduced hyphal damage, enhanced β-glucan exposure and altered vacuole homeostasis

**DOI:** 10.1371/journal.pone.0270676

**Published:** 2022-07-14

**Authors:** Sandeep Hans, Zeeshan Fatima, Aijaz Ahmad, Saif Hameed

**Affiliations:** 1 Amity Institute of Biotechnology, Amity University Haryana, Manesar, Gurugram, India; 2 Clinical Microbiology and Infectious Diseases, Faculty of Health Sciences, School of Pathology, University of the Witwatersrand, Johannesburg, South Africa; 3 Infection Control, National Health Laboratory Service, Charlotte Maxeke Johannesburg Academic Hospital, Johannesburg, South Africa; Louisiana State University, UNITED STATES

## Abstract

With a limited arsenal of available antifungal drugs and drug-resistance emergence, strategies that seek to reduce *Candida* immune evasion and virulence could be a promising alternative option. Harnessing metal homeostasis against *C*. *albicans* has gained wide prominence nowadays as a feasible antifungal strategy. Herein, the effect of magnesium (Mg) deprivation on the immune evasion mechanisms of *C*. *albicans* is demonstrated. We studied host pathogen interaction by using the THP-1 cell line model and explored the avenue that macrophage-mediated killing was enhanced under Mg deprivation, leading to altered cytokine (TNFα, IL-6 and IL10) production and reduced pyroptosis. Insights into the mechanisms revealed that hyphal damage inside the macrophage was diminished under Mg deprivation. Additionally, Mg deprivation led to cell wall remodelling; leading to enhanced β-1,3-glucan exposure, crucial for immune recognition, along with concomitant alterations in chitin and mannan levels. Furthermore, vacuole homeostasis was disrupted under Mg deprivation, as revealed by abrogated morphology and defective acidification of the vacuole lumen. Together, we demonstrated that Mg deprivation affected immune evasion mechanisms by: reduced hyphal damage, enhanced β-1,3-glucan exposure and altered vacuole functioning. The study establishes that Mg availability is indispensable for successful *C*. *albicans* immune evasion and specific Mg dependent pathways could be targeted for therapy.

## Introduction

*Candida albicans*, with mortality rates of 46–75%, causes life-threatening invasive infections every year globally [1]. Among hospital-acquired infections, it is the fourth most common cause [2], leading to life-threatening infections, particularly in immunosuppressed patients [3, 4]. Current antifungal drugs are active on a limited number of targets in *C albicans* and suffer from limitations, including the patient’s toxicity, a limited spectrum and the emergence of drug-resistant clinical isolates [5]. Hence, such antifungal interventions may be theoretically possible but are still far from being a way to eradicate yeast infections from the human microbiota. The alternative option, that could be more rewarding, is to seek a strategy that impairs *Candida* immune evasion; and thereby reducing its virulence.

*C*. *albicans* expresses virulence traits that confer the basis of a successful ability to evade immune responses and cause infectivity by the human fungal pathogen. This immune evasion by *C*. *albicans* could be attributed to several factors. For instance, the filamentation formed by yeast to hyphal transition is known to affect interactions with the host immune system [6]. Upon first encounter, macrophages can easily engulf the yeast forms, but have limitations with to phagocytose hyphal forms owing to their large sizes. Similarly, cell wall remodelling is another factor that facilitates immune evasion for *C*. *albicans*, where masking of crucial cell wall components such as β−1,3-glucan known to elicit the immune response occurs [7, 8]. In the inner cell wall layer of *C*. *albicans*, β-glucans are majorly masked from immune recognition by mannan fibrils located in the outer layer, and this ability to mask the immunogenic polysaccharide represents a key virulence factor [9]. The bud scars in yeast forms although they have exposed β−1,3-glucan sites for immune recognition, are lacking in hyphal forms, which leads to reduced macrophage recognition via C-type lectin receptor, dectin-1 [10]. Furthermore, upon ingestion by the macrophages, the cell wall remodelling induces a programmed cell death by pyroptosis that also facilitates fungal escape [11]. Additionally, a functional vacuole is also required for filamentation inside the macrophages, as the germ tube formation is known to be dependent on enlarged vacuole compartments, an ability again critical for immune evasion [12, 13]. Thus, the multifaceted fungal escape from the host immune response represents many vulnerable targets that could be utilized for antifungal therapy.

*C*. *albicans* must surmount the micronutrient (Fe, Zn, Mn and Mg) deprivation prevailing inside the hostile host environment for successful immune evasion [14]. These micronutrients because of their chemical ability to indulge in various futile reactions are not freely available, and instead, are withheld in bounded forms. Since both the host and the pathogen require these micronutrients for various enzymatic roles there is a continuous struggle for a limited access between host and pathogen. Magnesium (Mg) represents one such irreplaceable micronutrient that is indispensable for many reactions, involving cell signalling, ATP synthesis and nucleic acid metabolism [15]. Previous studies have already demonstrated that Mg deprivation affects the cellular circuitry governing the drug resistance of *C*. *albicans*, particularly revealing disrupted membrane homeostasis leading to improvement of the activities of membrane-targeting drugs and abrogation of efflux mechanisms by inhibition of ATP-binding cassette multidrug transporters [16–18]. To further comprehend the effect of Mg deprivation on host pathogen interaction, the present study elaborates on our understanding of the disruption of the immune evasion mechanisms of *C*. *albicans* under Mg deprivation and how it could be deployed in treatment strategies. We explored the avenue that Mg deprivation impairs *C*. *albicans* immune evasion by reduced hyphal damage, enhanced β-glucan exposure and altered vacuole homeostasis.

## Results

### Macrophage-mediated killing of *C*. *albicans* is enhanced under Mg deprivation

**Fig 1:**
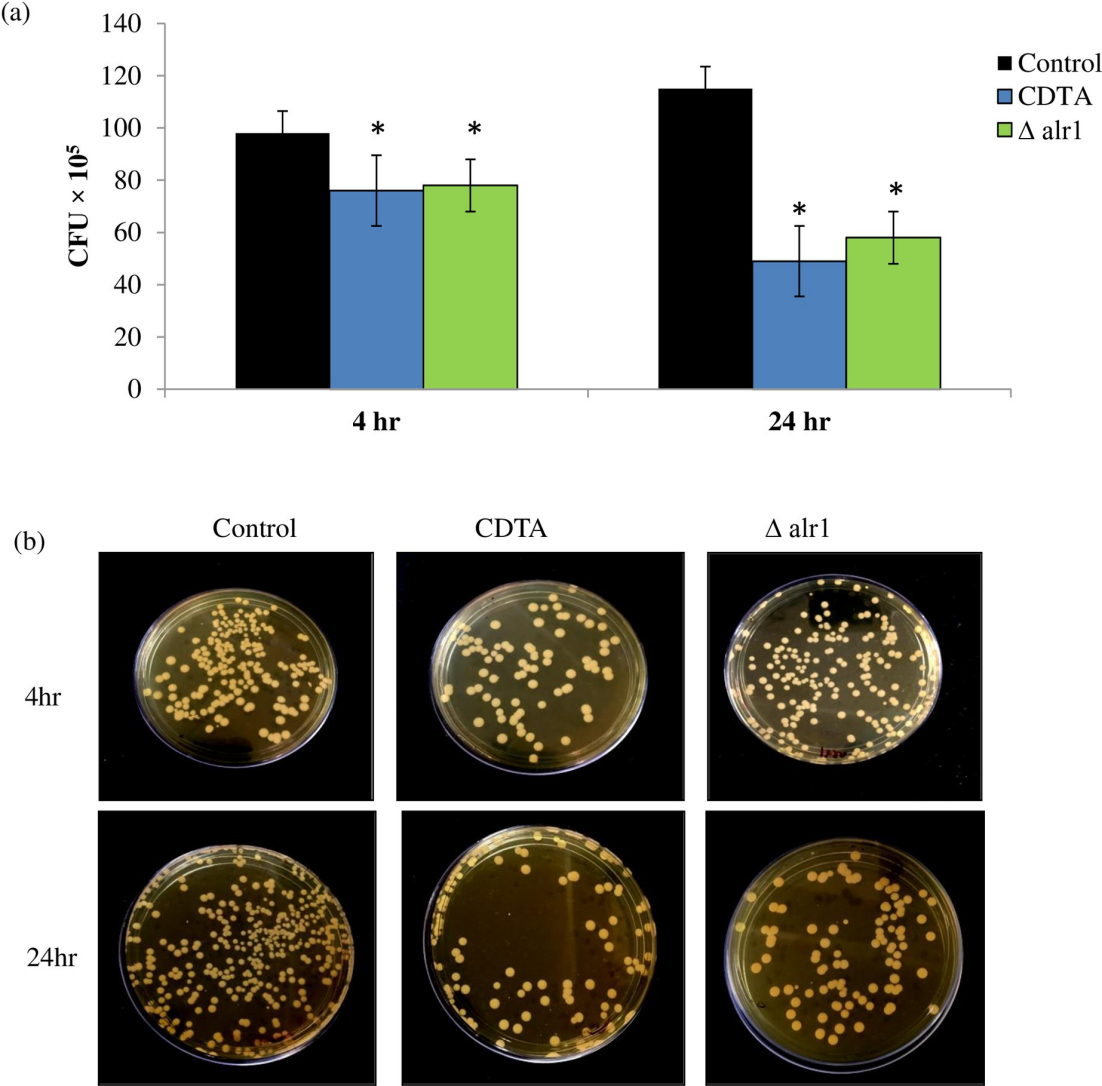
Macrophage killing under Mg deprivation. (**a**) Bar graph represents CFU log10^5^/ml on y-axis after 4- and 24-hours infection with control, CDTA treated and Δalr1 *C*. *albicans* depicting the killing of *C*. *albicans* by macrophages. Data are expressed as mean ± SD of three independent sets of experiments. *depicts *p* value < 0.05. (**b**) CFU image of control, CDTA treated and Δalr1 *C*. *albicans* after 4 and 24 hours infection.

Trans 1–2 diaminocyclohexane tetraacetic acid (CDTA), a well-known Mg chelator, was used to establish Mg deprivation. We used CDTA at a concentration 150 μg/ml that was sub-inhibitory enough without affecting the growth of *Candida* cells “S1 Fig” for all biochemical and phenotypic assays. Alr1p transporter is localized in plasma membrane and its expression is required mainly for Mg acquisition in *C*. *albicans*. Mg acquisition-defective *C*. *albicans* mutant (Δalr1) strain was also used to represent genetic inhibition of Mg availability to corroborate the observations of pharmacological suppression of Mg availability by CDTA. To examine whether the host immune response against *C*. *albicans* is affected under Mg deprivation, we exposed THP-1 cell derived macrophages to control, CDTA treated and Δalr1 *C*. *albicans*. The *Candida* growth inside the macrophage was indirectly determined by colony forming units per ml (CFUs/ml) over different time intervals (4 hours and 24 hours) to estimate the phagocytosis through the macrophage killing assay as described in methods. It was found that macrophage mediated killing was considerably enhanced under Mg deprivation at both the indicated time intervals, albeit more pronounced at 24 hours, with 11.5 × 10^6^ CFU/ml in control contrary to 49 × 10^5^ CFU/ml in CDTA treated and 58 × 10^5^ CFU/ml in Δalr1 “Fig 1A”. The images of control, CDTA treated and Δalr1 that were taken at the 4 hour and 24 hour intervals confirm the major differences in CFU “Fig 1B”. Hence, we concluded that Mg deprivation renders *C*. *albicans* less virulent in terms of enhanced susceptibly to macrophage-mediated killing.

### Pro-inflammatory cytokine production is stimulated under Mg deprivation

**Fig 2:**
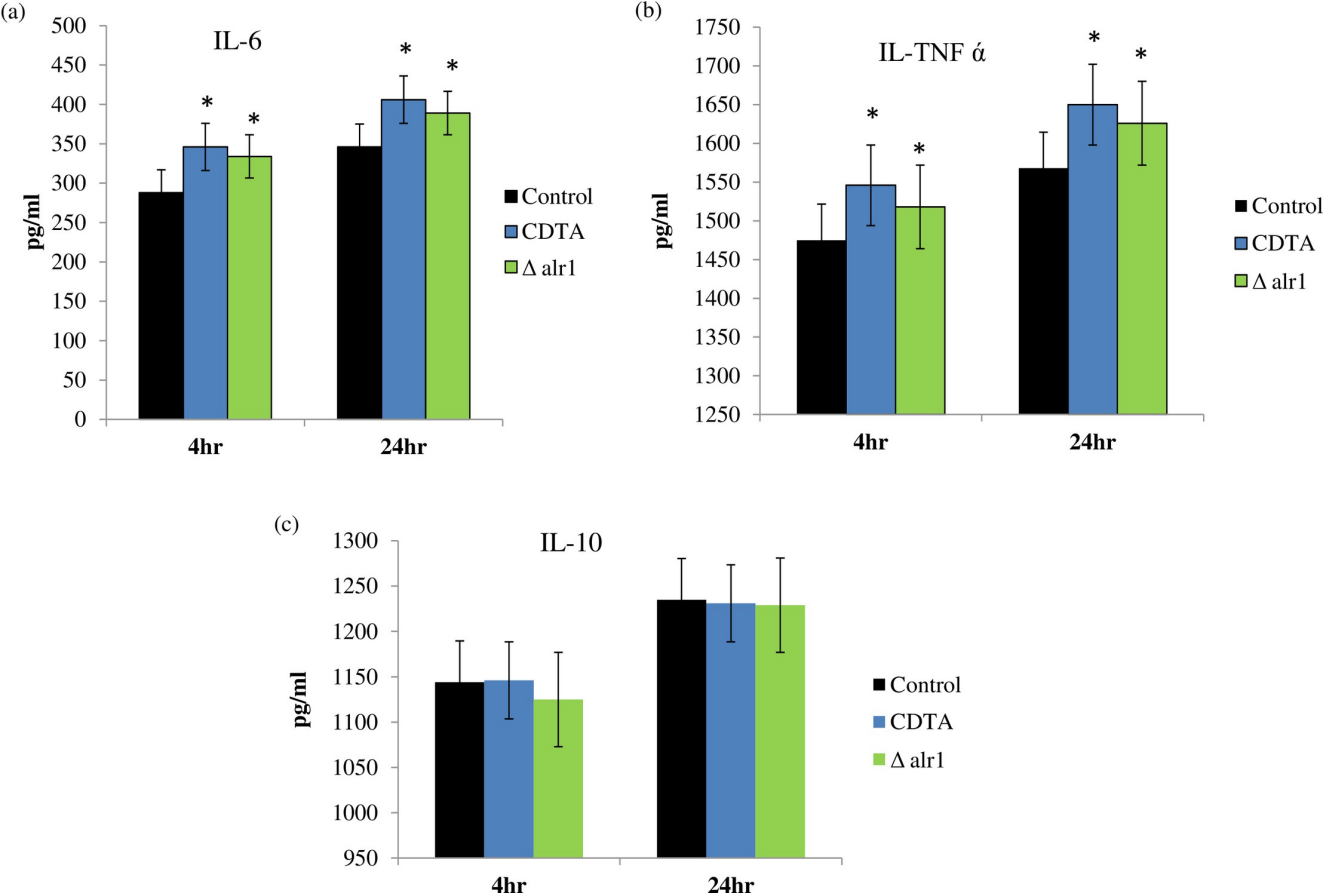
Cytokines stimulation under Mg deprivation. Bar graph represents cytokine levels measured in pg/ml on y-axis after 4- and 24- hours infection with control, CDTA treated and Δalr1 *C*. *albicans*. Supernatants were collected after infection and analyzed with specific ELISA for cytokines (**a**) IL-6 (**b**) TNFα (**c**) IL-10. Cytokine titters were calculated by reference to standard curves generated by the four parameters logistic curve-fit method. The results represent the mean of three independent experiments, * depicts *p* value < 0.05.

Next, we aimed to assess the ability of *C*. *albicans* to stimulate cytokine production under Mg deprivation by using THP-1 cell lines. We checked the levels of both the pro-inflammatory and anti-inflammatory cytokine secretion of THP-1 cells under Mg deprivation. After completion of control, CDTA treated and Δalr1 *C*. *albicans* infection of 4 hours and 24 hours, THP-1 cell supernatants were collected and proinflammatory cytokine (IL-6 and TNFα) levels and one anti-inflammatory cytokine (IL-10) level were determined by ELISA. We observed enhanced production of proinflammatory cytokines, IL-6 “Fig 2A” and TNFα “Fig 2B” in THP-1 infected cells with CDTA treated and Δalr1 *C*. *albicans*, albeit more pronounced after 24 hours, and no significant difference in anti-inflammatory cytokine IL-10 levels “Fig 2C”. This suggested that Mg deprivation stimulated enhanced production of pro-inflammatory cytokines.

### Intracellular pyroptosis of macrophages is inhibited under Mg deprivation

**Fig 3:**
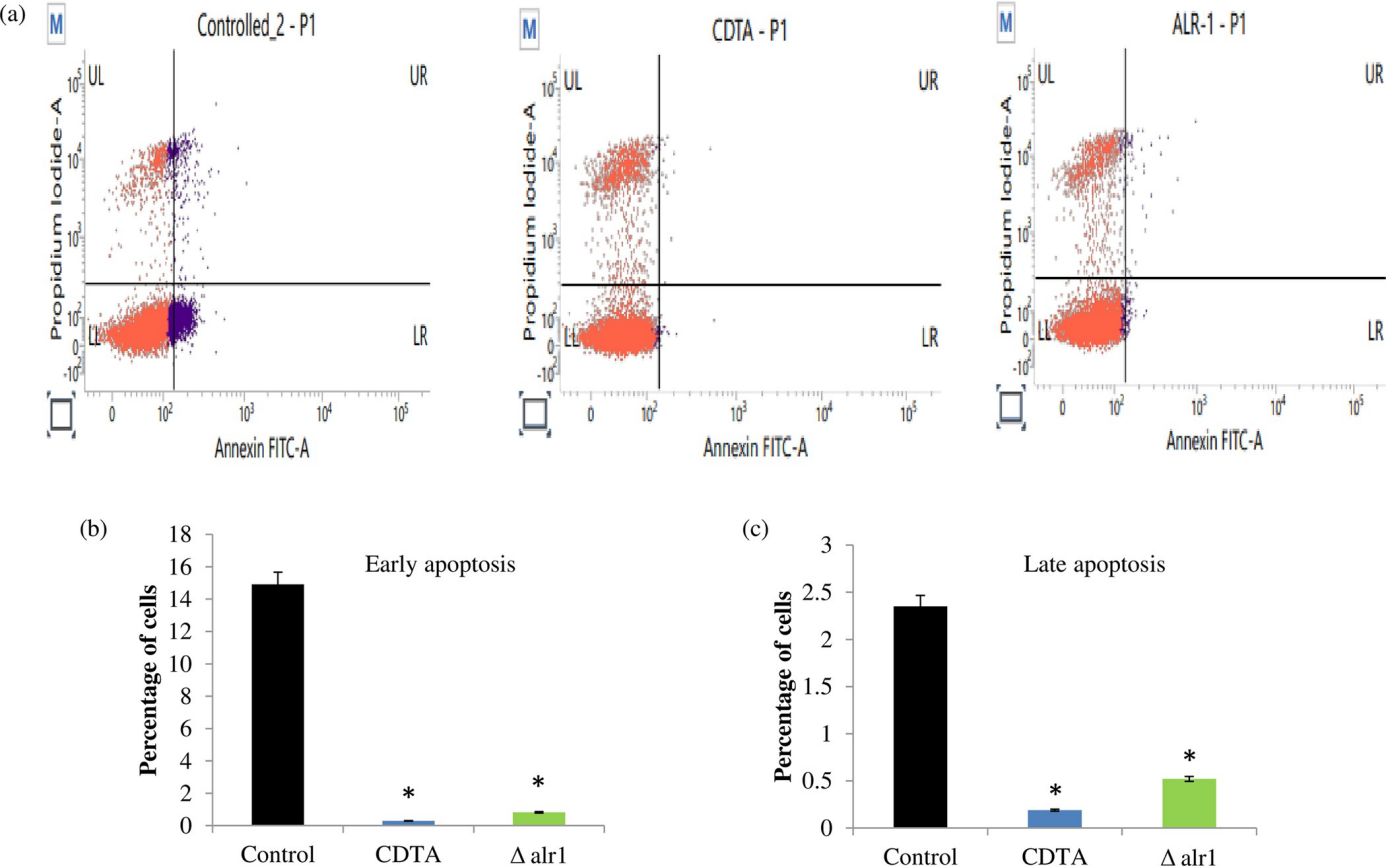
Pyroptosis of macrophages under Mg deprivation. (**a**). Flow-cytometry analysis of Annexin V–positive cells in control, CDTA treated and Δalr1 *C*. *albicans*. The representative bar graphs represent percentage of Annexin V or propidium iodide positive cells for (**b**) early and (**c**) late pyroptosis respectively.

Subsequently, we studied the pyroptosis of the 24 hour control, CDTA treated and Δalr1 *C*. *albicans* infected THP-1 derived macrophage cells through flow cytometry. After the infection, cells were collected at 24 hours, stained with annexin V-FITC and Propidium iodide (PI). We found that CDTA treated and Δalr1 infected THP-1 cells induced negligible pyroptosis compared to control cells “Fig 3A”. Both early “Fig 3B” and late apoptosis “Fig 3C” was significantly decreased under Mg deprivation. This result reinforces the observation that Mg deprivation confers a defect in induction of pyroptosis, leading to enhanced immune recognition.

### Hyphal damage inside macrophages is diminished under Mg deprivation

**Fig 4:**
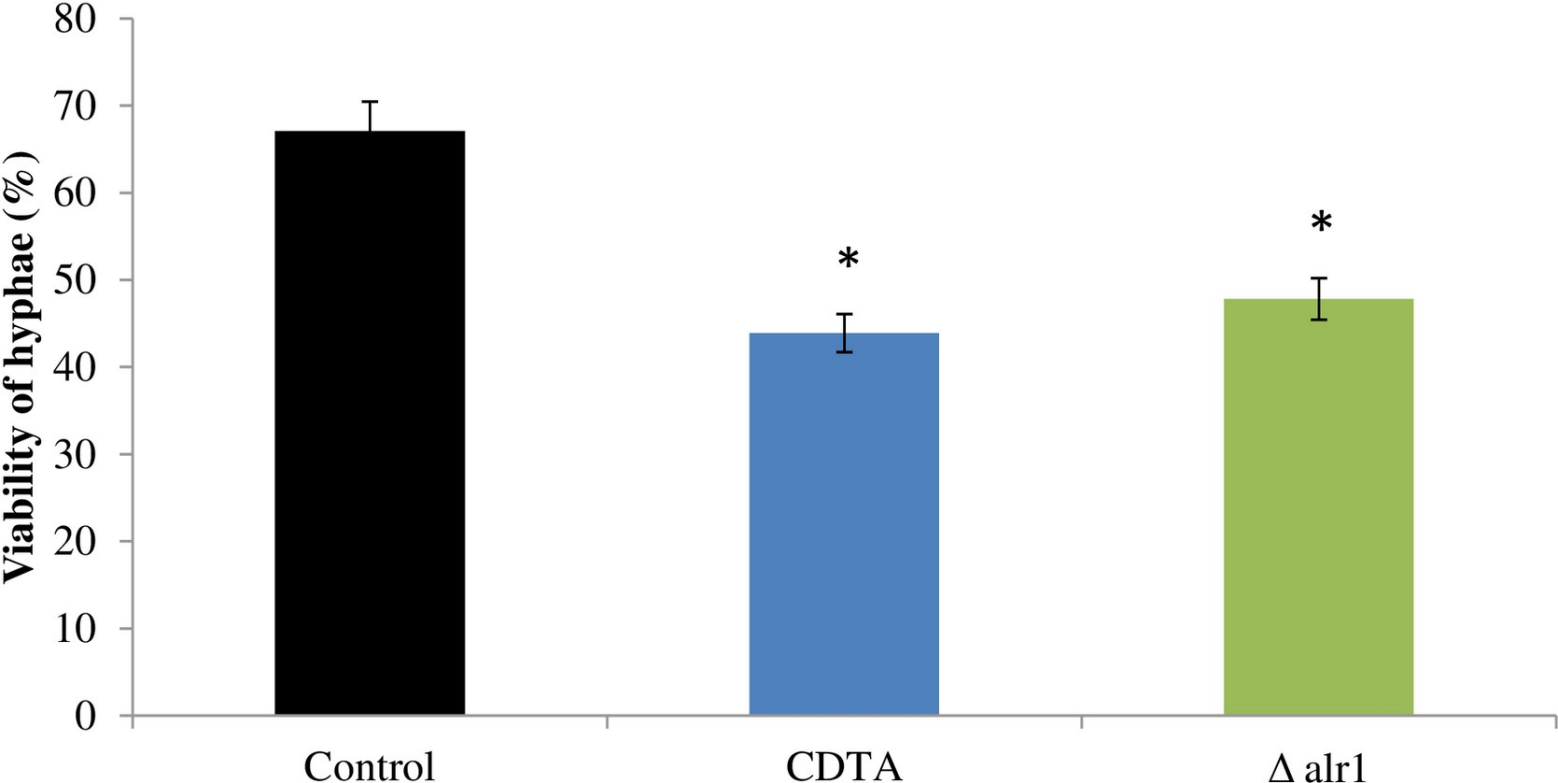
Hyphal damage under Mg deprivation. Percentage of hyphal damage expressed as viability induced by control, CDTA treated and Δalr1 *C*. *albicans* incubated at an E/T ratio of 5:1 THP-1 for 24 hours. The results represent the mean of three independent experiments, * depicts *p* value < 0.05.

Next, we sought to explore the possible mechanisms underlying immune recognition under Mg deprivation conditions. We studied the yeast-to-hyphal transition under Mg deprivation, which is a critical virulence trait responsible for macrophage lysis once internalized, and also promotes fungal escape. For this, we tested infected THP-1 cells with control, CDTA treated and Δalr1 *C*. *albicans* for their ability to form hyphae within the macrophages, by estimating the hyphal damage through a colorimetric assay estimating the viability of hyphae as described in methods. We observed that viability of hyphae in CDTA treated and Δalr1 cells was reduced by 26% and 17% respectively, as compared to control cells, which displayed robust filamentation “Fig 4”. This result confirmed that macrophage-induced *C*. *albicans* hyphal damage is reduced under Mg deprivation.

### β-1,3-glucan exposure is enhanced under Mg deprivation

**Fig 5:**
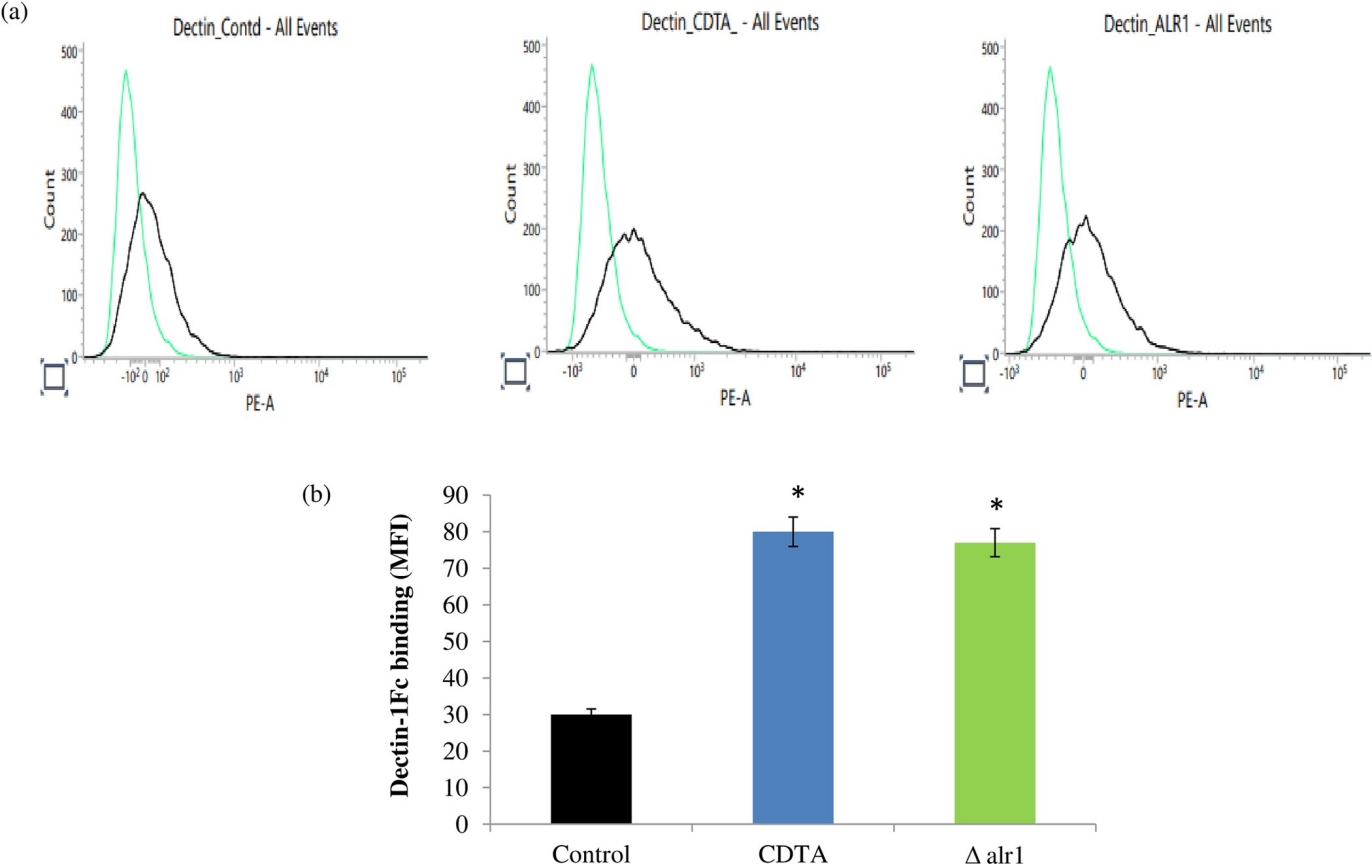
β-1,3 glucan exposure under Mg deprivation. (**a**) Representative flow cytometry plots display β1,3-glucan exposure by dectin-1-Fc bindings in control, CDTA treated and Δalr1 *C*. *albicans*. (**b**) Representative bar graphs depicting cumulative data of MFI of Dectin-1-Fc binding from 3 independent experiments.

Filamentation inside the macrophages is coupled to cell wall remodelling, where β-1, 3-glucan masking is another important factor that plays a major role in determining the immune response of the host [19]. We determine the β -1, 3-glucan unmasking of control, CDTA treated and Δalr1 using dectin-1 Fc labelling that enables detection of exposed β-1,3-glucan in *C*. *albicans*, and the mean fluorescence intensities (MFI) depicting this binding was quantified by flow cytometry. We observed that CDTA and Δalr1 treated *C*. *albicans* cells displayed two-fold higher MFI as compared to control cells “Fig 5”. This result suggested that β -1, 3-glucan exposure is enhanced (unmasking) under Mg deprivation.

### Cell wall composition is altered under Mg deprivation

**Fig 6:**
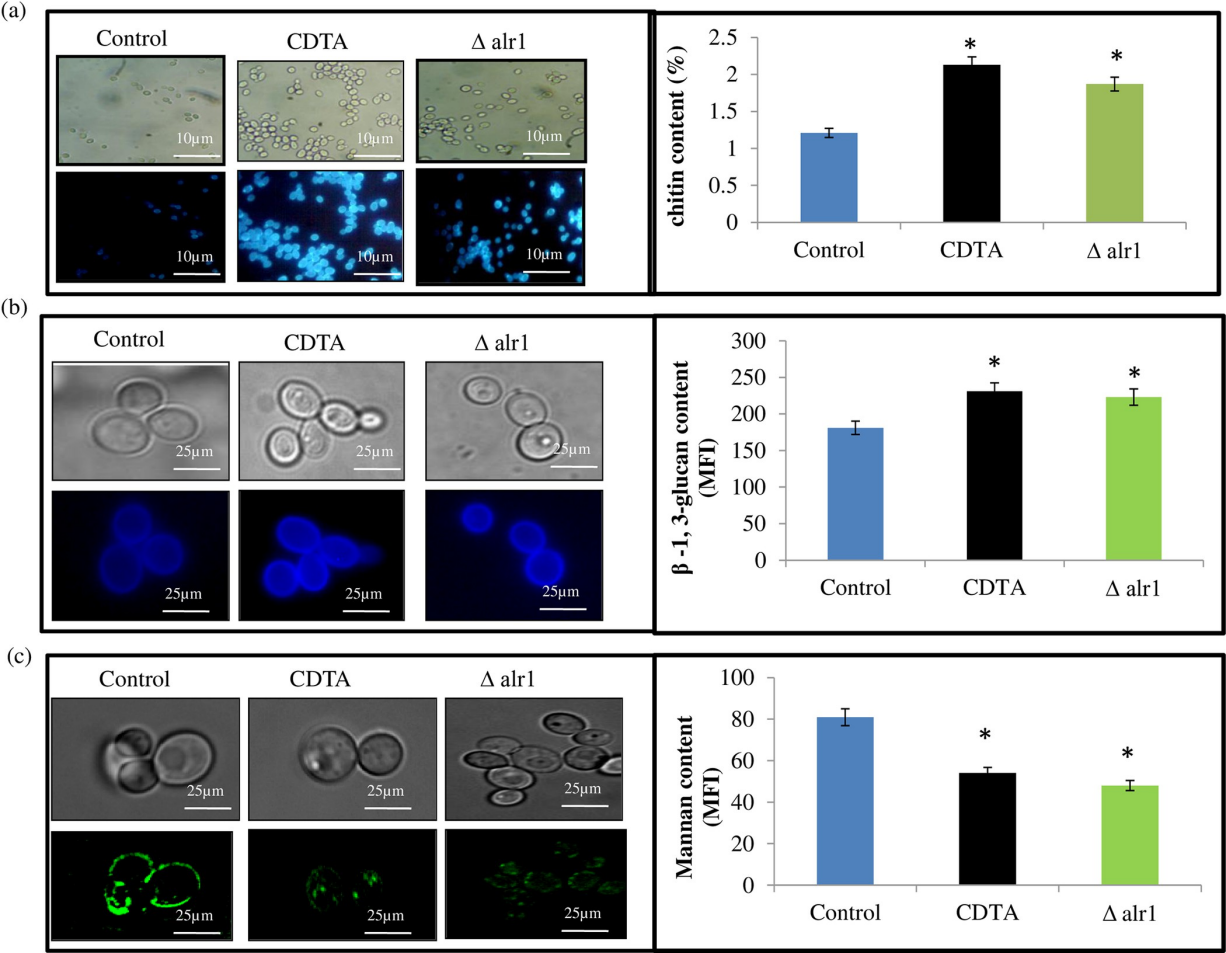
Changes in cell wall composition under Mg deprivation. Left panels depict representative fluorescent micrographs for control, CDTA treated and Δalr1 *C*. *albicans* stained for (**a**) chitin (CFW), (**b**) β1,3-glucan (AB) and (**c**) mannan (ConA). Scale bar = 25 μm. Right panels depict the levels of (a) chitin (b) β1,3-glucan and (c) mannan measured in control, CDTA treated and Δalr1 *C*. *albicans* cells. The results represent the mean of three independent experiments, *depicts *p* value < 0.05.

Furthermore, to examine whether the cell wall composition of *C*. *albicans* is altered under Mg deprivation, we estimated the major components of the cell wall (chitin, β -1, 3-glucan and mannan) more intricately. For this, control, CDTA treated and Δalr1 *C*. *albicans* cells were stained with CFW (for chitin), AB (for β -1,3-glucan) and concanavalin A (for mannan) and observed the changes in the cell wall components by using a fluorescence microscope. We observed that chitin “Fig 6A” and β -1, 3-glucan “Fig 6B” staining with their respective dyes were enhanced in CDTA and Δalr1 cells, contrary to control cells, while mannan stained by ConA was diminished “Fig 6C” under Mg deprivation. Furthermore, with the help of these specific dyes, all the three components were also quantitatively measured, and the results were concomitant with the qualitative estimation (“Fig 6” right panels). Together, we inferred that Mg deprivation led to an increase in chitin and β -1, 3-glucan contents while decreasing in mannan content.

### Sensitivity to cell wall perturbing agents is enhanced under Mg deprivation

**Fig 7:**
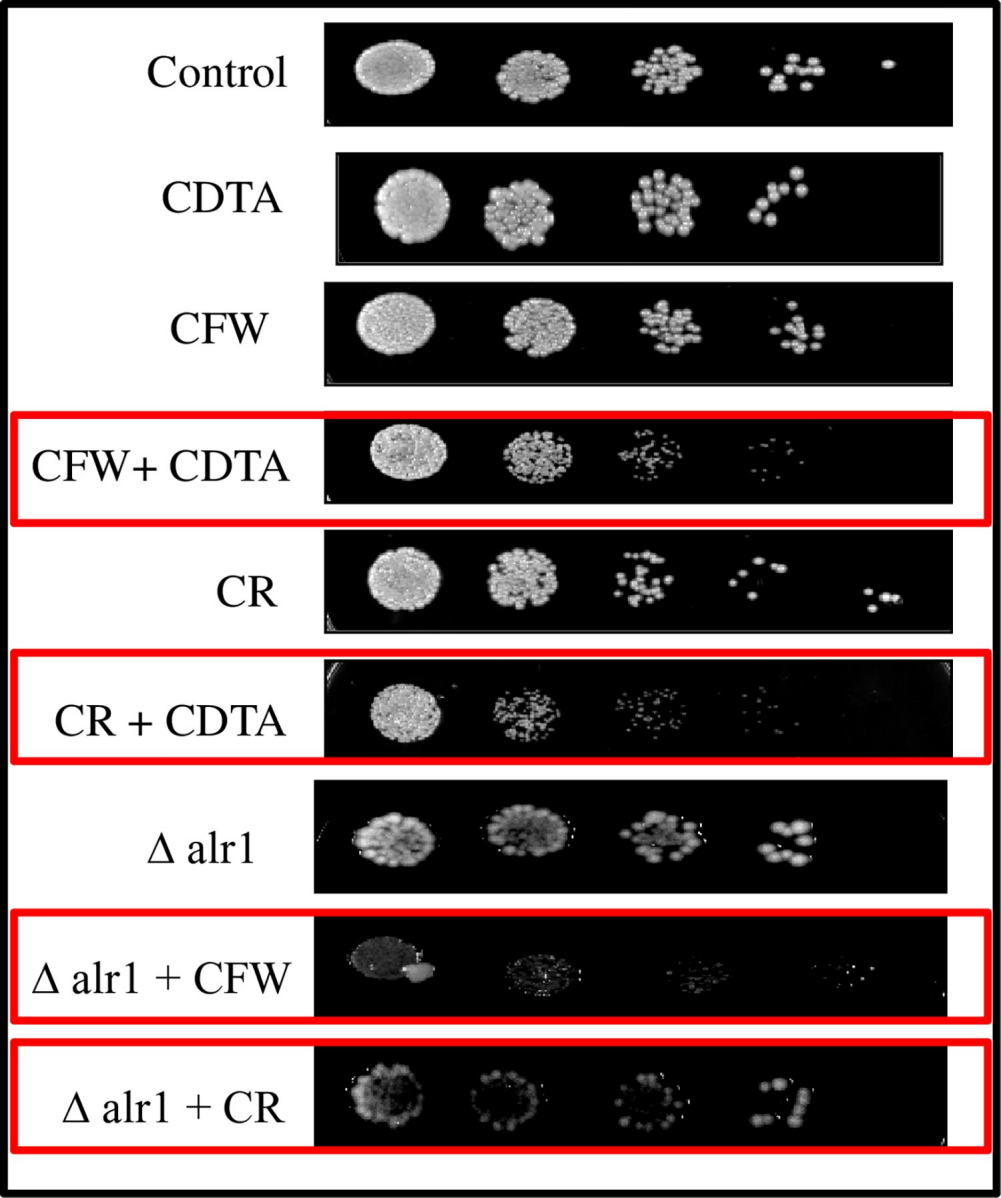
Sensitivity to cell wall perturbing agents under Mg deprivation. Spot assay of control, CDTA treated and Δalr1 *C*. *albicans* in presence of CR (10μg/ml) and CFW (10μg/ml).

Next, we hypothesized that cell wall remodelling under Mg deprivation may affect the susceptibility of *C*. *albicans* to cell wall disrupting agents. For this, we tested the sensitivity of control, CDTA treated and Δalr1 *C*. *albicans* cells to Calcofluor white (CFW) and Congo red (CR). The phenotypic susceptibility spot assay revealed that susceptibility was moderately enhanced under Mg deprived conditions in the presence of both CFW and CR “Fig 7”. Additionally, we estimated the cell sedimentation rate of *C*. *albicans* under Mg deprivation to assess cell ability to form clumps owing to cell wall remodelling. We found that the rate of sedimentation was considerably enhanced in CDTA treated and Δalr1 cells “S2 Fig”. Overall, Mg deprivation induces changes to cell walls, rendering them susceptible to cell wall damaging agents.

### Vacuolar homeostasis is disrupted under Mg deprivation

**Fig 8:**
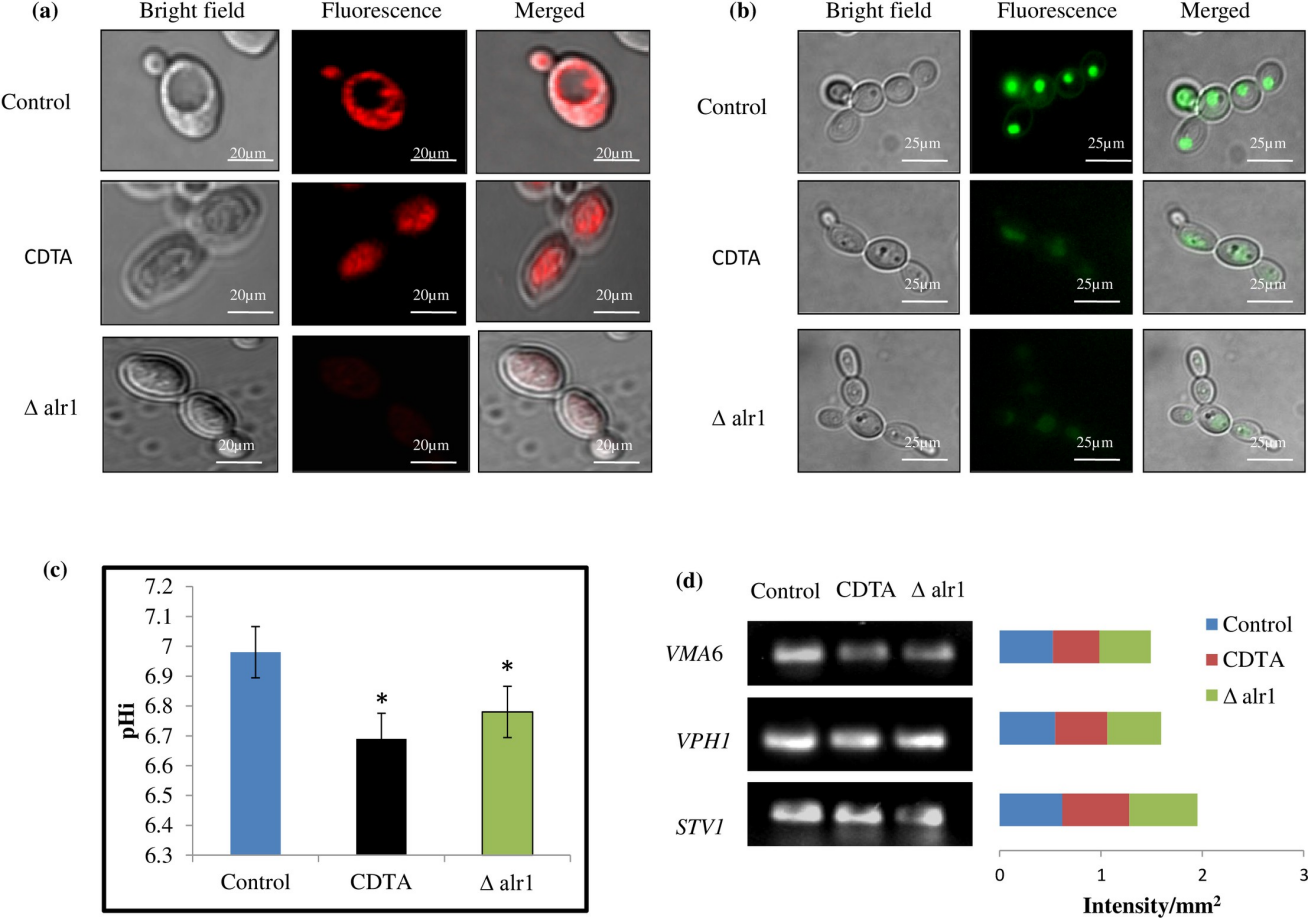
Vacuolar homeostasis under Mg deprivation. (**a**) Vacuolar morphology of control, CDTA treated and Δalr1 *C*. *albicans* observed using FM4-64 staining. Scale bar = 20 μm. (**b**) Vacuolar pH of control, CDTA treated and Δalr1 *C*. *albicans* observed using quinacrine staining. Scale bar = 25 μm. **(c)** Cytosolic pHi under Mg deprivation. Measurement of Intracellular pH (pHi) in control, CDTA treated and Δalr1 *C*. *albicans*. The bar graph depicts mean of pHi and pHi ± SD of three independent sets of experiments, *depicts p value < 0.05. **(d)** RT-PCR under Mg deprivation. Left panel represents gel images of differentially expressed genes (*VMA6*, *VPH1* and *STV1*) in *C*. *albicans*. Right panel depicts the quantitation (density expressed as Intensity/mm^2^) of the respective transcripts. Image J software was used to measure the intensity of each band.

Filamentation inside the macrophages is also associated with the functional vacuole in *C*. *albicans* [12]. Hence, we checked the functionality of *C*. *albicans* vacuole by observing vacuole morphology and acidification. To check the vacuole morphology, we used FM4-64, a lipophilic dye that is actively endocytosed and specifically binds to the vacuole membrane. We observed that in control cells, FM4-64 was gathered at the vacuolar membrane, exhibiting the typically ring-staining pattern depicting the normal morphology, contrary to that of the CDTA treated and Δalr1 cells, where FM4-64 was localized to the vacuolar lumen and diffusely distributed, depicting a defect in morphology “Fig 8A”. To check the vacuolar acidification, we used quinacrine dye, a weak base that fluoresces only under acidic conditions inside the vacuole when protonated, as it cannot come out of the vacuole. We observed that in control cells quinacrine exhibited high fluorescence, implying that the vacuole had a normal acidic environment, contrary to the CDTA treated and Δalr1 cells where there was less fluorescence, depicting elevated vacuolar pH “Fig 8B”. These observations led us to believe that Mg deprivation abrogates vacuole homeostasis by disrupting morphology and acidification.

### Cytosolic pH of *C*. *albicans* is decreased under Mg deprivation

Abrogated vacuolar acidification prompted us to ascertain the cytosolic pHi, which might have decreased due to accumulation of H^+^ under Mg deprivation. Thus, we ascertain whether the disrupted vacuolar homeostasis under Mg deprivation leads to any inhibition of V-ATPase activity because of which H^+^ movement across the vacuole membrane is inhibited. We observed that Mg deprivation causes H^+^ accumulation as depicted by decreased pHi in CDTA treated and Δalr1 *C*. *albicans* cells “Fig 8C”. This result suggested that Mg deprivation leads to decrease in pHi.

### *VMA6* expression is moderately reduced under Mg deprivation

Lastly, we examined the V- ATPase subunit complex transcripts (*VMA6*, *VPH1* and *STV1*) under Mg deprivation; under similar growth conditions as used for other analyses by semiquantitative RT-PCR. We found a moderate decrease in the transcript level of *VMA*6 gene, with no considerable difference in *VPH1* and *STV1* transcripts in CDTA treated and Δalr1 *C*. *albicans* cells contrary to control “Fig 8D”.

## Discussion

Since elimination of fungal infections, particularly those caused by *C*. *albicans*, is far from becoming reality; studies that aid in decreasing the virulence and immune evasion of *C*. *albicans* could be a promising alternative. *C*. *albicans*, during the process of pathogenesis, encounters various stress conditions which must be overcome for its sustenance. One such hostile condition which *C*. *albicans* must surmount is the nutritional immunity adopted as a defence strategy by the human host [20]. Under nutritional immunity, the host withholds significant micronutrients which are crucial for the growth of the pathogens and the host. Mg is one such crucial micronutrient that is required for variety of cell physiological processes [15], in addition to the recently discovered roles governing drug-resistance circuitry in *C*. *albicans* [16–18]. The present study is an attempt to expose various mechanisms and targets that are vulnerable under Mg deprivation during the process of encounter with host immune recognition cells for evasion and fungal escape.

*C*. *albicans* escapes killing by host immune cells by various mechanisms crucial for fungal survival inside the host. Hence, firstly, we studied the host-pathogen interaction in response to Mg deprivation. We found that macrophage-mediated killing of *C*. *albicans* was enhanced under both (pharmacological and genetic) tested Mg deprived conditions, as revealed by fewer CFU/ml “Fig 1”. Additionally, we observed stimulation of pro-inflammatory cytokines (IL-6 and TNFα; “Fig 2”) which are pre-requisites for fungal clearance [21]. Once engulfed by professional phagocytes such as macrophages, *C*. *albicans* induces an inflammatory cell death programme known as pyroptosis that helps the fungus to evade the host defence [22]. In our study, we found that Mg deprivation causes negligible pyroptosis to host cells contrary to the control cells “Fig 3”. These observations provided sufficient evidence to establish the fact that immune evasion of *C*. *albicans* was impaired under Mg deprivation and that Mg availability is indispensable for fungal escape.

Further, insights into the mechanisms disrupted under Mg which could possibly be responsible for the observed impaired immune evasion were studied. *C*. *albicans*, being a dimorphic fungus, has two morphological forms, namely yeast and hyphae. These interconvertible states have major implications on the interaction of *C*. *albicans* with immune cells [6]. It is well known that macrophages can internalize yeast cells but are not able to phagocytose the hyphal forms, owing to their large sizes [23]. Therefore, hyphal morphogenesis in *C*. *albicans* is an indicator of virulence, as most of the studied mutant strains which are defective in hyphal morphogenesis are avirulent [24–27]. In the present study, we observed that hyphal damage owing to filamentation is less under Mg deprivation, as revealed by reduced percentile hyphal viability “Fig 4”. This corresponds with the previous study which demonstrated the inhibition of yeast-to-hyphal transition under Mg deprivation in tested *in vitro* hyphal inducing conditions [16]. Furthermore, functional hyphal morphogenesis is a key aspect in inducing macrophage pyroptosis, as the mutants defective in hyphal morphogenesis are unable to induce macrophage pyroptosis [12]. Hence, we could hypothesize that defective filamentation could be one of the possible reasons behind impaired immune evasion mechanisms of *C*. *albicans* under Mg deprivation.

Apart from the role of yeast-to-hyphal transition in avoiding macrophage internalization, the filamentation process is also coupled to cell wall remodelling, that leads to the masking of inner cell wall components such as β-1,3-glucan, crucial for eliciting immune recognition [10, 12]. β-1,3-glucan is a key pathogen-associated molecular pattern (PAMP), which is recognized by pathogen-recognition receptors (PRR) such as dectin-1, located on the macrophages [28, 29]. Therefore, we examined the effect of Mg deprivation on β-1,3-glucan exposure. Interestingly, we could observe enhanced exposure of β-1,3-glucan in the presence of dectin-1-Fc under Mg deprivation “Fig 5”. Further, we assessed the cell wall composition intricately and found that the levels of chitin and β-1,3-glucan were enhanced, while mannan content was considerably reduced “Fig 6”. This observation is particularly fitting, as mannan is the outermost covering in the cell wall of *C*. *albicans*, while chitin and β-1,3-glucan are buried inside [30]. The decreased mannan as observed in the present study may have facilitated the unmasking of the inner cell wall components chitin and β-1,3-glucan, which stimulated the immune recognition under Mg deprivation. The above compositional changes in the cell wall of *C*. *albicans* under Mg deprivation also had an effect on its susceptibility to cell wall perturbing agents CFW and CR (Fig 7A) and cell sedimentation rates “S2 Fig”. Thus, overall, we concluded that cell wall remodelling mainly triggered by greater exposure of β-1,3-glucan, could be another possible reason for enhanced immune recognition under Mg deprivation.

*C*. *albicans* vacuole is an acidic cubicle having numerous enzymes involved in hydrolysis and forms an important storage site for cellular metabolites. *C*. *albicans* morphogenesis is also dependent upon the dynamics of functional vacuoles [31]. Mutants of a fungus with noteworthy vacuolar defects are unable to grow and cause lethality in a mouse with disseminated disease, as mutants of the vacuole gene *VPS1* have an attenuated ability to kill the macrophages [12]. This also implies that some of the vacuole functions are indispensable for *C*. *albicans’* survival within the macrophages. Thus, we intended to study the functionality of the vacuole under Mg deprivation, in terms of morphology by vacuole specific dye FM4-64 and acidification by a weak base, quinacrine. We found that both the morphology and the acidification of the vacuole was abrogated under Mg deprivation, as revealed by FM4-64 and quinacrine staining respectively “Fig 8A & 8B”. Abrogated vacuolar acidification also prompted us to estimate the cytosolic internal pH (pHi), which we found to be expectedly less owing to cytosolic accumulation of H^+^ “Fig 8C”.

V-ATPase is a multi-subunit evolutionary conserved proton pump, which is responsible for maintaining the pH gradient across the endomembrane and vacuole and the endocytic pathways in the cell [32]. A defect in the V-ATPase disrupts many processes necessary for infection-countering host immunity, maintaining organelle pH and tolerance of antifungal drugs [33]. The endocytic recycling of the Wsc1 cell wall stress sensor is dependent on V-ATPase-mediated vacuole lumen acidification [34]. Lastly, we studied the transcript levels of three V-ATPase genes, *VMA6*, *VPHI* and *STV1*, to ascertain whether the abovementioned changes in vacuole functioning could also be regulated at transcriptional level. The V_1_ subcomplex that governs ATP hydrolysis, coupled to proton translocation, is encoded by one of the isoforms *VMA6* [35]. The V_0_ subcomplex of V-ATPase that governs proton translocation inside the vacuole lumen is encoded by two isoforms *VPH1* and *STV1* [36]. In the present study we could detect a moderate decrease in *VMA6* transcript under Mg deprivation “Fig 8D”. Thus, we concluded from these observations that disruption of vacuolar homeostasis under Mg deprivation should also be the reason for impaired immune evasion. We further speculate that disruption of vacuole homeostasis could be owing to inhibition of V-ATPase activity; however, this needs to be validated by further research. Additionally, it is known that decreased ergosterol synthesis also has an impact on the functioning of vacuole [37]. Hence, we further extrapolate that abrogated vacuole homeostasis under Mg deprivation could also partly be caused by decreased ergosterol content observed previously [16].

## Methods

### Growth media and strains used

The YPD medium comprising yeast extract 1% (w/v), peptone 2% (w/v), and dextrose 2% (w/v) was used to culture the *C*. *albicans* SC5314 strain (wild type control). 2% (w/v) agar was added to the media for the agar plates. Mg acquisition-defective *C*. *albicans* mutant (Δalr1) strain was provided as a gift [38] to support the observations by CDTA treatment.

### Macrophage infection and killing assay

THP-1 cells were cultured in Roswell Park Memorial Institute (RPMI) 1640 medium with 10% Fetal Bovine Serum (FBS). The cells were treated with 15 nM Phorbol 12-myristate 13-acetate (PMA) for 48 hours and then washed three times. In YPD media, the control, CDTA-treated, and Δalr1 *C*. *albicans* were grown to log phase. Prior to infection, each *Candida* strain 1 ml culture was pelleted for 2 minutes, reconstituted in RPMI 1640, vortexed for 2 minutes, and passed through a syringe (26 gauge needle) 2–3 times. Macrophages were infected with *C*. *albicans* for 4 hours to achieve a 5:1 infection ratio (pathogen: host), then treated with amikacin (200 g/ml). The cells were resuspended in RPMI supplemented with 10% FBS, after being rinsed with PBS. After 24 hours of infection supernatants were collected, aspirated, and monolayers were gently washed three times with PBS before being lysed with 0.5% Triton–X to determine the number of CFUs. The lysates were serially diluted in triplicate and plated on agar plates, to determine CFUs as previously described [21, 39].

### Cytokine analysis

Supernatants of the control and 4- and 24- hour infection with CDTA treated and Δalr1 *C*. *albicans* from above experiment were either stored at -20°C or tested for levels of cytokines TNFα, IL-6 and IL-10 using an enzyme linked immunosorbent assay (ELISA) kit according to manufacturer’s (ABTS, Peprotech, USA) instructions [40].

### Intracellular pyroptosis

THP-1 cells’ pyroptosis was measured using an Annexin-FITC staining kit (R&D Systems, Minn., and USA) according to the manufacturer’s instructions, and flow cytometry (BD FACS (USA) using Suite software) was used to interpret the results. THP-1 cells infected with control, CDTA-treated, and Δalr1 were detached with cold PBS (4°C), pelleted at 300 g for 10 minutes, and washed in 1 ml PBS. The Annexin-FITC reaction mixture was prepared in 100 μl of binding buffer according to the instructions, then added to the samples and gently vortexed. After labelling, the cells were incubated in the dark for 15–30 minutes with the reaction mixture before being fixed in 4% paraformaldehyde. Prior to acquisition, an extra 400 μl of binding buffer was added. FACScan was used to collect the labelled cells, which were subsequently examined using Cellquest software [41].

### Hyphal damage

A colorimetric assay utilising 3-(4,5-dimethylthiazol 2-yl)-2,5-diphenyltetrazolium bromide (MTT) was performed on 12-well culture plates to assess the hyphal damage of control, CDTA treated, and Δalr1 following incubation with THP-1 cell. THP-1 cells were lysed with 0.5% sodium deoxycholate and the wells were rinsed with PBS after a 24-hours incubation period. After that, 1 ml RPMI 1640 supplemented with 1% MTT was added to the wells, and the plates were incubated for 3 hours at 37°C with a 5% CO_2_ concentration. 1 ml acidic isopropanol (95 percent isopropanol and 5% 1 N HCl) was added to the wells before detection, and the plates were incubated until the blue colour faded. A spectrophotometer was used to measure the absorbance of the supernatant at a wavelength of 550 nm (VSI Electronics, India). The following formula was used to determine the viability of the hyphae:

$$\mathrm{Viability}\;\mathrm{of}\;\mathrm{hyphae}=\frac{0D550\;\mathrm{sample}}{0D550\;\mathrm{control}}\times 100$$

where OD_550_ sample was the absorbance measured for the hyphae incubated with THP-1 cell and OD_550_ control was the absorbance measured for the hyphae incubated without THP-1 cell [39, 42].

### Flow cytometry analysis of β-1,3-glucan exposure

5 × 10^6^ cells of control, CDTA treated and Δalr1 *C*. *albicans*, were cultured overnight at 30°C in YPD broth and washed with PBS, incubated with 10 μg of Dectin-1 Fc, or PBS for 2 hours at 4°C. *Candida* cells were washed with PBS, followed by incubation with pre-conjugated donkey antihuman IgG (Jackson ImmunoResearch) for 30 minutes. *Candida* cells were then again washed with PBS, fixed with BD FACS (USA) using Suite software [40, 43].

### Chitin estimation

The chitin content was estimated by measuring the absorbance of glucosamine released by acid hydrolysis as described previously [40, 44]. Briefly, the overnight-grown cultures (control, CDTA treated and Δalr1 *C*. *albicans*) were inoculated in YNB medium supplemented with 2% glycerol, and 6 × 10^6^ yeast cells were harvested. The cells were then washed, suspended in sterile distilled water, and disrupted with 0.5-mm glass beads in a homogenizer. Cell debris was washed five times with 1 M NaCl. The cell wall was extracted in SDS-MerOH extraction buffer (50 Mm Tris, 2% sodium dodecyl sulphate, 0.3 M β-mercaptoethanol, 1 mM EDTA, pH of 8.0) at 100°C for 10 minutes. After 1 hour, the absorbance was measured at 520 nm using a spectrophotometer. The glucosamine concentration in each sample was determined from a standard curve of 0–0.3 mg/ml of glucosamine. Subsequently, chitin content was calculated as the percentage of cell wall dry weight.

### β-1,3-glucan estimation

Aniline blue (AB) staining was used to quantify β—1,3-glucan as previously described with slight modifications [35, 45]. Overnight cultures of control, CDTA treated, and Δalr1 were grown to log phase, washed twice, and suspended in TE buffer (10 mM Tris pH 8.0, 1 mM EDTA) to achieve a final OD_600_ of 0.5, with 1 M NaOH added. The solution was then incubated in a water bath at 80°C for 30 minutes, which allowed the β—1,3-glucan to solubilize. After that, 2.1 ml AB mix (0.03 percent AB, 0.18 M HCl, and 0.49 M glycine/NaOH, pH 9.5) was added, and the tubes were incubated for 30 minutes at 50°C, followed by another 30 minutes at ambient temperature to allow the fluorochrome to react and for subsequent decolorization. A fluorescence plate reader (Agilent, USA) and cart eclipse software were used to measure fluorescence. The wavelength of excitation was 400 nm, and the wavelength of emission was 460 nm.

### Staining of cell wall components

To determine the cell wall components, 2.5 × 10^6^ exponential cells of control, CDTA treated and Δalr1 were grown and fixed with 4% paraformaldehyde for 30 minutes in PBS. After three washes with PBS, fixed cells were stained with 50 μg/ml Concavalin A (ConA) for mannan, 100 μg/ml of AB for β-1,3-glucan, 5 μg/ml of Calcofluor white (CFW) for chitin, followed by incubation at room temperature for 30 minutes. Fluorescence-stained cells were seen by a fluorescence microscope (Nikon, Japan) at 100X magnification. Images were taken using NIS element software [46].

### Spot assay

The spot assay was performed using a previously described method [17] with the control, CDTA treated and Δalr1 *C*. *albicans*. Briefly, 5 mL of five-fold serially diluted yeast cultures (OD_600_ = 0.1) were spotted onto YPD plates. The growth difference was assessed after 48 hours at 30°C.

### Relative sedimentation

Cell sedimentation was measured spectrophotometrically as described previously [47]. Briefly, overnight culture of control, CDTA treated and Δalr1 *C*. *albicans* were inoculated to 0.1 OD_600_ and allowed to grow till the OD_600_ reaches 1.0. The OD_600_ from each culture were measured at regular intervals of 5 minutes till 1 hour. Sedimentation rates were measured by recording the differences in growth from zero time point to a 60 minute per unit time interval, and calculated as described in “S2 Fig”.

### Vacuolar morphology and fluorescence imaging

The control, CDTA treated, and Δalr1 *C*. *albicans* were cultured overnight in YPD. The cells were then rinsed in 1X PBS and resuspended in fresh YPD for 4 hours of growth. Then 40 μM FM4-64 was added and the cells were further incubated for 15 minutes at 30°C, then washed and resuspended in fresh YPD, incubated for 60 minutes at 30°C, and images were obtained using a fluorescent microscope at 100x magnification [35, 48].

### Quinacrine staining

1ml yeast cells from YPD medium overnight control, CDTA treated, and Δalr1 *C*. *albicans* were extracted to harvest the cells by centrifugation for 5 minutes at 3,800 g and resuspended in 1ml of YPD medium. 10 μl of the Quinacrine solution (10 mg/ml; Molecular Probes) was then added and incubated for 5 minutes. A fluorescence microscope was used to acquire images at a magnification of 100× [35, 48, 49].

### Intracellular pH (pHi)

Intracellular pH was measured as described previously [47, 50]. Mid-log phase control, CDTA treated and Δalr1 *C*. *albicans* cells grown in YPD medium were harvested and washed twice with distilled water. Cells (0.1g) were suspended in 5 ml solution containing 0.1 M KCl and 0.1 mM CaCl_2_. Control, CDTA treated and a Δalr1 *C*. *albicans* cell were added to the suspension and pH was adjusted to 7.0 in each group. Following incubation for 30 min at 37°C with constant shaking at 200 g, pH was again adjusted to 7.0. The change in pH of suspension was followed on pH meter with constant stirring. The value of external pH at which nystatin permeabilization induced no further shift, was taken as an estimate of pHi.

### RT-PCR

RNA isolation from *C*. *albicans* was performed using TRIzol and RNeasy Mini Kit with DNase (QIAGEN) treatment [16, 51]. Cells were diluted in 50 mL of fresh YPD broth at an OD_600_ of 0.1 (10^6^ cells/ ml) in the absence (control), presence of CDTA and Δalr1 grown at 30°C to an OD_600_ of 1.0. For reverse transcription PCR (RT-PCR), cDNA was synthesized using a Revert Aid TM H Minus cDNA Synthesis Kit (Invitrogen, Waltham, MA) from the isolated RNA. The synthesized cDNA product (2 ml) was directly used for PCR amplification (50 ml) using gene-specific forward and reverse primers “S1 Table”. The amplified products were gel electrophoresed and the densities of bands (for genes of interest) were measured and quantified by Image J software.

### Statistical analysis

All experiments were performed in three independent biological replicates (n = 3). The results were reported as mean ± standard deviation (SD) and analyzed by using Student’s t test in which P<0.05 was considered as statistically significant.

## Conclusion

The discovery of new antifungal drugs is a cumbersome process, and the available limited repertoire of current antifungal drugs suffer from limitations, including host toxicity and the emergence of drug-resistant clinical isolates. Under the current pressing circumstances; instead of trying to eradicate *Candida* infections from the human microbiota, it would be worthwhile to seek strategies that can reduce *Candida* immune evasion, thereby reducing its virulence. The present study has demonstrated that Mg deprivation affects several targets that are important for *Candida* virulence and immune recognition. The application of the present strategy in aiding the current therapeutics will lie in the fact that we need to target specific Mg dependent pathways (transporters or regulators) of *C*. *albicans* and design inhibitors against them. For instance, Alr1p transporter is localized in plasma membrane and its expression is required mainly for Mg acquisition in *C*. *albicans*. Additionally, there is no homolog of Alr1p in human and is specific to the *Candida* cells. We can design an inhibitor which targets Alr1p without affecting host to help combating *Candida* infections. In pursuit of the above alternative option, the present study supports evidence that the immune evasion mechanisms of *C*. *albicans* could be impeded under Mg deprivation, and this strategy could be implemented in treatment strategies.

## Supporting information

S1 Fig Growth curve of *C*. *albicans* under Mg deprivation. Graph shows the growth of control, CDTA (150μg/ml) treated and Δalr1 *C*. *albicans*. *y*-axis depicts O.D_600_ nm of *C*. *albicans* growth. x-axis depicts time in hours.
(DOCX)

S2 Fig Cell sedimentation rates of *C*. *albicans* under Mg deprivation. Left panel shows absorbance of control, CDTA treated and Δalr1 *C*. *albicans* depicted on *y* axis with respect to time (minutes) on *x*-axis. Right panel shows sedimentation rates per minutes on y-axis for control, CDTA treated and Δalr1 *C*. *albicans*, considered by estimating the difference in absorbance from 0 till 30 minutes per unit time interval. The results represent the mean of three independent experiments, * depicts *p* value < 0.05.
(DOCX)

S1 Table List of primers used in the study for RT-PCR. (DOCX)
