# Supplementary material for: Magnesium impairs Candida albicans immune evasion by reduced hyphal damage, enhanced β-glucan exposure and altered vacuole homeostasis
Source: PLoS One. 2022 Jul 14;17(7):e0270676. doi: 10.1371/journal.pone.0270676 (PMC9282612; doi:10.1371/journal.pone.0270676)
Supplement: S1 Fig — Graph shows the growth of control, CDTA (150μg/ml) treated and Δalr1 C. albicans. y-axis depicts O.D600 nm of C. albicans growth. x-axis depicts time in hours. (DOCX) [file pone.0270676.s001.docx]

**S1 Fig: Growth curve of *C. albicans* under Mg deprivation**. Graph shows the growth of control, CDTA (150µg/ml) treated and Δalr1 *C. albicans. y*-axis depicts O.D_600_ nm of *C. albicans* growth. x-axis depicts time in hours.
