# Supplementary material for: Magnesium impairs Candida albicans immune evasion by reduced hyphal damage, enhanced β-glucan exposure and altered vacuole homeostasis
Source: PLoS One. 2022 Jul 14;17(7):e0270676. doi: 10.1371/journal.pone.0270676 (PMC9282612; doi:10.1371/journal.pone.0270676)
Supplement: S2 Fig — Left panel shows absorbance of control, CDTA treated and Δalr1 C. albicans depicted on y axis with respect to time (minutes) on x-axis. Right panel shows sedimentation rates per minutes on y-axis for control, CDTA treated and Δalr1 C. albicans, considered by estimating the difference in absorbance from 0 till 30 minutes per unit time interval. The results represent the mean of three independent experiments, * depicts p value < 0.05. (DOCX) [file pone.0270676.s002.docx]

**S2 Fig:** **Cell sedimentation rates of *C. albicans* under Mg deprivation.** Left panel shows absorbance of control, CDTA treated and Δalr1 *C. albicans* depicted on *y* axis with respect to time (minutes) on *x*-axis. Right panel shows sedimentation rates per minutes on y-axis for control, CDTA treated and Δalr1 *C. albicans*, considered by estimating the difference in absorbance from 0 till 30 minutes per unit time interval. The results represent the mean of three independent experiments, * depicts *p* value < 0.05.
