# Supplementary material for: Magnesium impairs Candida albicans immune evasion by reduced hyphal damage, enhanced β-glucan exposure and altered vacuole homeostasis
Source: PLoS One. 2022 Jul 14;17(7):e0270676. doi: 10.1371/journal.pone.0270676 (PMC9282612; doi:10.1371/journal.pone.0270676)
Supplement: S1 Table — (DOCX) [file pone.0270676.s003.docx]

| **Sr. No.** | **Gene name** | **Primer Sequence** |
| --- | --- | --- |
|  | *VMA6* | **F-** CTTTAACATCGACTACGGGT  **R-** CAGCTATGTAGCTTTCAATTC |
|  | *STV1* | **F-** AGAAGCAGTATTCCGTTCAGC  **R-** ATTAAACCGGTGGGACCAAA |
|  | *VPH1* | **F-** AGACTTTCCGAAAATAGCGTG  **R-** CAACCCAATGCAATCTTAACG |

**S1 Table : List of primers used for RT–PCR in the study.**
